# Factors associated with cervical screening coverage: a longitudinal analysis of English general practices from 2013 to 2022

**DOI:** 10.1093/pubmed/fdad275

**Published:** 2023-12-26

**Authors:** Sean Urwin, Stephanie Gillibrand, Jennifer C Davies, Emma J Crosbie

**Affiliations:** Health Organisation, Policy and Economics Group, School of Health Sciences, University of Manchester, Manchester M13 9PL, UK; Health Organisation, Policy and Economics Group, School of Health Sciences, University of Manchester, Manchester M13 9PL, UK; Gynaecological Oncology Research Group, Division of Cancer Sciences, Faculty of Biology, Medicine and Health, University of Manchester, Manchester M13 9PL, UK; Department of Obstetrics and Gynaecology, St Mary's Hospital, Manchester Academic Health Science Centre, Manchester University NHS Foundation Trust, Manchester M13 9WL, UK; Gynaecological Oncology Research Group, Division of Cancer Sciences, Faculty of Biology, Medicine and Health, University of Manchester, Manchester M13 9PL, UK; Department of Obstetrics and Gynaecology, St Mary's Hospital, Manchester Academic Health Science Centre, Manchester University NHS Foundation Trust, Manchester M13 9WL, UK

**Keywords:** screening, general practice, cancer

## Abstract

**Background:**

Cervical cancer remains an important global public health concern. Understanding the factors contributing to a decline in screening uptake in high-income countries is fundamental to improving screening rates. We aimed to identify general practice and patient characteristics related to cervical screening coverage in England between 2013 and 2022.

**Methods:**

We analyzed a panel of 59 271 General Practice (GP)-years from 7881 GP practices. We applied correlated random effects regression to examine the association between cervical screening uptake and a rich set of GP practice workforce, size, quality and patient characteristics.

**Results:**

Our results show a decline in overall screening rates from 2013/14 to 2021/22 from 77% to 72%. We find GP workforce and list size characteristics are strongly related to screening rates. An increase in 1 FTE Nurse per 1000 patients is related to a 1.94 percentage point increase in cervical screening rates. GP practices located in more deprived areas have lower screening rates.

**Conclusions:**

GP workforce and patient characteristics need to be considered by decision-makers to increase screening rates. The implementation of self-sampling screening methods could help address some of the current barriers to screening, including lack of healthcare staff and facilities.

## Introduction

As the fourth most common cancer and leading cause of cancer death among women globally, cervical cancer is an important public health concern.^1^ In 2020, the World Health Organisation announced a strategy to reduce the impact of cervical cancer.^2^ A crucial component of this strategy is timely and effective cervical screening.^3^^,^^4^ Despite this, two-thirds of women aged 30–49 worldwide have never been screened.^5^ In England, there has been an overall decline in cervical screening rates,^6^ yet ~60% of Clinical Commissioning Groups and nearly half of local authorities have failed to take action.^7^

The COVID-19 pandemic resulted in a suspension of screening invitations in England between April and June 2020.^8^ There is a fear that pre-existing inequities in cervical screening uptake were further exacerbated by the pandemic, and experts have called for additional screening capacity as well as risk-based modeling to improve coverage.^9^^,^^10^

Reviews of screening coverage from low and middle-income countries conclude that cultural, logistical and practical barriers^11^ as well as structural and socio-economic factors^12^ all influence cervical screening rates. In high-income countries, socio-economic factors including income and region are associated with screening rates,^13^^,^^14^ as well as age, marital status and employment.^15^

There is limited evidence from England on the factors associated with screening coverage. Evidence from qualitative research emphasize personal barriers including, previous bad experiences, practical barriers (childcare, time-constraints, appointment times), perception of low-risk, fear, pain and discomfort and lack of information and awareness^16–20^ which may impact the propensity to undergo screening. Quantitatively, patient barriers to screening are associated with socio-demographic characteristics, including ethnicity.^21^ Two UK based studies found that General Practice (GP) with a higher proportion of female patients aged 25–49 years old, and those with a higher proportion of ethnic minority residents were more likely to have low screening rates.^22^^,^^23^ However, the current evidence is limited to cross-sectional rather than longitudinal data,^22^^,^^23^ and studies that use individual level data do not typically analyze healthcare characteristics.

In this study, we analyze at the general practice level, practice characteristics including access, quality and deprivation, as well as patient characteristics including age, ethnicity and geographic factors that may influence screening coverage in England. We contribute to the literature in three ways. First, by providing a contemporary assessment of the predictors of cervical screening between 2013 and 2022 of all practices in England. Second, we analyze a range of different practice and patient characteristics not previously explored in the literature such as the number and type of staff within GP practices. Third, we apply a correlated random effects technique that allows us to estimate our practice and patient characteristics over time in tandem.

## Methods

### Data sources

We combined information from five sources including: The Quality and Outcomes Framework (QOF); GP workforce; GP patient lists; the GP Patient Survey (GPPS); and the 2011 census for England.

### Outcome

We obtained our measure for cervical screening rates from GP practice level QOF data available from National Health Service (NHS) Digital.^24^ QOF is a pay for performance mechanism that rewards GP practices based on the achievement of clinical and non-clinical goals. Cervical screening indicators form part of the public health domain of the QOF. They are proportional measures where the numerator is those who meet the criteria and the denominator is all those eligible for cervical screening. Our measure applies to all eligible women between 25 and 64 years old. We provide additional details of the indicator in appendix section A1.1.

### Practice characteristics

We analyzed a set of patient, workforce, accessibility and quality measures of GP practices based on previous evidence that has explored cervical screening rates^22^ and the availability of general practice related data.

Information about GP workforce is publicly available from NHS Digital.^25^ We used the total number of full-time equivalent (FTE) general practitioners, nurses and administrative staff from each annual September release of these data from 2013 to 2021. We calculated for each staff type the total number of FTE per 1000 patients of the general practice. We chose these staff types as previous evidence has found general practice workforce skill-mix is related to primary care outcomes.^26^ We assigned workforce characteristics to cervical screening rates that cover the same financial year.

Information on the characteristics of a general practice’s patient list is publicly available from NHS Digital.^27^ We obtained the total number of patients registered at a GP practice in thousands.

We used several measures of general practice quality and a measure of practice accessibility from publicly available sources such as the QOF and the GPPS. Our first measure of practice quality used the clinical domain of the QOF which was the proportion of total achieved points out of the maximum available. Our second measure of practice quality is from the GPPS. The GPPS is a national survey distributed biannually until 2015 and then annually thereafter.^28^ The practice quality measure captures the proportion of patients who rated their overall experience of their practice as very good. Our measure of practice accessibility captures the proportion of patients who were very satisfied with the opening time of their practice. All our measures from the GPPS are aggregated to a practice level for each financial year. The aggregation used weights to account for non-response and survey design.

We used the income deprivation domain which captures deprivation relating to low income from the Index of Multiple Deprivation (IMD) and is publicly available.^29^^,^^30^ We assign the 2015 income deprivation measure to the financial years 2013/14 and 2014/15 and the 2019 income deprivation measure to the financial years 2015/16 to 2021/22. We measure income deprivation of the GP practice location in quintiles.

### Patient characteristics

We used the proportion of each general practice’s patient list that are female and meet the eligibility age criteria of between 25 and 64 years old. We used snapshot information from September of each financial year between 2013/14 and 2021/22.

To obtain the rurality and ethnic mix of a general practice’s patient list we used published information on the total number of patients in each Lower Super Output Area (LSOA) from yearly snapshots in September provided by NHS Digital.^27^ We calculated the proportion of a practice list that reside in an LSOA defined as rural according to the ONS.^31^ We combined the LSOA patient totals with LSOA ethnicity information from the 2011 census in England. We assumed the ethnic mix of each LSOA remained constant throughout the time period of analysis and applied these proportions to practice list LSOA data. We therefore obtained, at the practice level, the proportion of a practice that were in a minority ethnic group which according to the 2011 census metagroups are: mixed ethnicity; Black; Asian; and other. We provide details of the eighteen ethnic groups and five metagroups in appendix section A1.2.

The predictors that capture the age-gender, ethnicity and rurality composition of the practice list from September of each year is assigned to the cervical screening rate that covers the same financial year.

### Statistical analyses

We had an initial dataset of 8020 GP Practices which is equivalent to 64 353 GP practice-year observations, shown in Figure A1 in the appendix. We removed GP practices which ever had a list size lower than 1000 patients. GP practices of this smaller size may not be representative of typical GP practices and may also be indicative of a closing GP practice. This sample restriction is commonplace in the literature analyzing associations between primary care characteristics.^32^^,^^33^ We achieved a final sample of 59 218 GP practice-year observations from 7881 GP practices across nine financial years with complete cases in the outcome and predictors.

We used a correlated random effects regression technique^34^^,^^35^ where our model specification is:


$${y}_{it}={\beta}_0+{\beta}_1{PR}_{it}+{\beta}_2{PA}_{it}+{\tau}_t+{a}_i+{\varepsilon}_{it}$$

Where ${y}_{it}$ is the cervical screening rate for practice $i$ in financial year $t$, ${PR}_{it}$ is the vector of practice characteristics, ${PA}_{it}$ is the vector of slow-moving patient characteristics across $t$. ${\beta}_1$ and ${\beta}_2$are the respective coefficient vectors, ${\tau}_t$ is the financial year indicators, ${a}_i$ the practice fixed effect, ${\varepsilon}_{it}$ the idiosyncratic error and ${\beta}_0$ is the model intercept.

Our approach allows the unobserved practice effect, ${a}_i$, to depend on observed practice characteristics:


$$ {a}_i=\rho +\varnothing \overline{PR_i}+{u}_i $$

Where $\overline{PR_i}$ is the time average of the practice characteristics, ${u}_i$is the independent portion of the practice effect and $\rho$ is a constant. This permits correlation between the time-invariant error term ${a}_i$ and the average of the time varying practice variables $\overline{PR_i}$, assuming all time-invariant heterogeneity is identified by the average of the time varying practice variables. We chose this technique as our patient characteristics such as ethnicity, age and deprivation have limited within practice variation and would produce imprecise coefficients. Therefore, correlated random effects allows the interpretation of year-to-year changes in practice characteristics on year-to-year changes in screening rates while controlling for unobserved time-invariant practice heterogeneity. We report standard errors clustered at the practice level to account for correlation within practices in the error term.

We used a Hausman-type specification test to provide evidence for or against the correlated random effects approach. If we reject the null that $\overline{PR_i}$ is jointly insignificant, then this test indicates correlated random effects is preferred over a random effects specification.

We conducted three sensitivity checks: (i) including small GP practices that ever have a list size with ˂1000 patients; (ii) excluding the financial year 2020/21 as this year may have been affected by lockdowns imposed as a result of the COVID-19 pandemic and (iii) analyzing a balanced panel of practices that are present across all nine financial years.

We used Stata 17 for all data analysis.

### Public engagement

The results of the study were presented to the study’s Advisory Group, made up of diverse members of the public. The group shared reflections about the difficulty of accessing and booking appointments in primary care post-pandemic which it was felt hindered access to screening (alongside primary care more widely). The importance of the role of healthcare professionals during the screening process was also mentioned, in respect to booking the appointments, and undergoing the examination, where it was important that patients were made to feel comfortable. Clinical members of the project team also highlighted workforce issues as barriers to screening rates. It was noted that largely, only female practitioners undertook the training (due to patient preference for female practitioners to do the test) to conduct screening and therefore this limited the workforce available to offer smears.

## Results

**Fig. 1:**
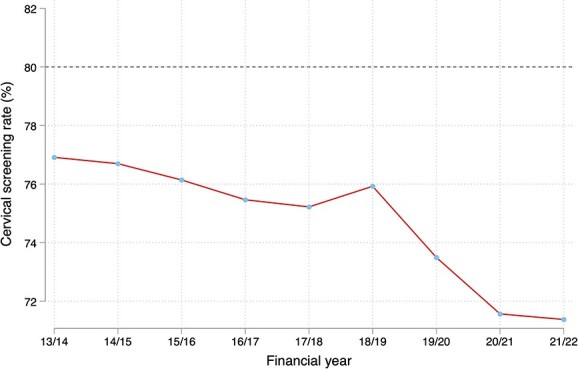
Cervical screening rates in England. *Note*: The statistics in this figure are the authors calculations from QOF data. Each practice is weighted according to the total eligible individuals for screening.

Figure 1 shows the average cervical screening rate across General Practices in England between the financial years 2013/14 and 2021/22. The dashed line denotes the 80% target that is viewed as the ideal across high-income countries. The average cervical screening rate across England has been declining since 2013/14 from 77% to under 72% in 2021/22.

Table 1 presents summary statistics of our outcome and predictor measures. Across our sample, the average cervical screening rate is 75.13%. The practices we analyze have, on average, 0.27 FTE nurses and 1.14 FTE administrative staff per 1000 patients and an average list size of 8370 patients. 27.08% of practices are located in the most deprived income deprivation quintile. Practices have, on average, 26.35% of their list size, that are female and between 25 and 64 years old. Between standard deviations (variation across practices) when compared to overall standard deviations are larger for the patient than practice characteristics. Within standard deviation (variation within practices) are larger for practice than patient characteristics when compared to the overall standard deviation. For example, the within and overall standard deviation is 0.08 and 0.15 for FTE nurses, respectively.

**Table 1:** Summary statistics

Variable	Percentage (or mean)	Overall Standard deviation	Between Standard deviation	Within Standard deviation
**Outcome:**				
Cervical screening rate	(75.13)	7.23	6.68	3.24
**Practice characteristics:**				
**Workforce:**				
Full-time equivalent GPs (per 1000 patients)	(0.58)	0.25	0.22	0.15
Full-time equivalent nurses (per 1000 patients)	(0.27)	0.15	0.14	0.08
Full-time equivalent admin staff (per 1000 patients)	(1.14)	0.37	0.35	0.21
**Size:**				
List size (000’s)	(8.37)	5.26	4.92	1.73
**Quality:**				
Overall patent experience: very good	45.54	15.04	13.20	7.44
QOF clinical achievement	95.35	6.61	6.24	4.26
**Access:**				
Patient satisfaction with opening hours: very good	50.74	29.31	13.83	27.17
**Income deprivation quintile of practice location:**				
1st quintile (least deprived)	11.77	32.22	30.91	7.75
2nd quintile	16.32	36.96	34.53	12.11
3th quintile	20.71	40.52	38.15	12.98
4th quintile	24.12	42.78	41.23	12.08
5th quintile (most deprived)	27.08	44.44	44.23	8.10
**Patient characteristics:**				
**Age group:**				
25–64 years old	26.35	2.58	2.55	0.51
**Ethnic group:**				
Minority (mixed, Asian, Black, other)	16.28	19.45	19.74	0.61
**Geographical:**				
Rurality	17.71	32.62	31.71	1.57
Practice year observations	59 218			
Practices	7881			

*Note:* The cervical screening rate mean drops to 74.78% (SD: 7.47) once practices are weighted by their list size.

**Fig. 2:**
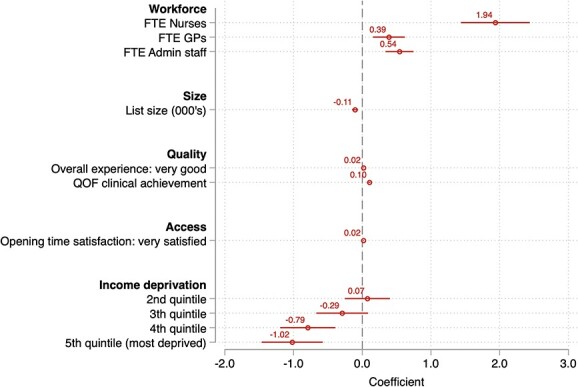
Association between practice characteristics and cervical screening rates. *Notes:* The chi-squared statistic from the Hausman-type specification test is 9.76 (*P* < 0.01) indicating the correlated random effects specification is preferred. 95% confidence intervals are presented.

Figure 2 presents results on practice characteristics estimated with correlated random effects regression. An increase in 1 FTE nurse per 1000 patients is related to a 1.94 (95% CI: 1.44–2.44) percentage point increase in the screening rate, on average. An increase in a practice list size by 1000 patients is related to, on average, a 0.11 (95% CI: 0.13–0.06) percentage point decrease in the screening rate. Higher practice quality and greater accessibility is related to higher screening uptake. A 1 percentage point increase in the QOF clinical achievement score is associated with a 0.11 (95% CI: 0.10–0.13) percentage point higher cervical screening uptake. However, the overall experience and opening time satisfaction coefficients are smaller at 0.02 (95% CI: 0.01–0.02) for both coefficients. Practices in the most deprived national quintile of income deprivation have a 1.02 (95% CI: 1.46–0.57) lower screening rate than those in the least deprived quintile, on average. The chi-squared statistic from the Hausman-type specification test is 9.76 (*P* < 0.01) indicating the correlated random effects specification is preferred.

**Fig. 3:**
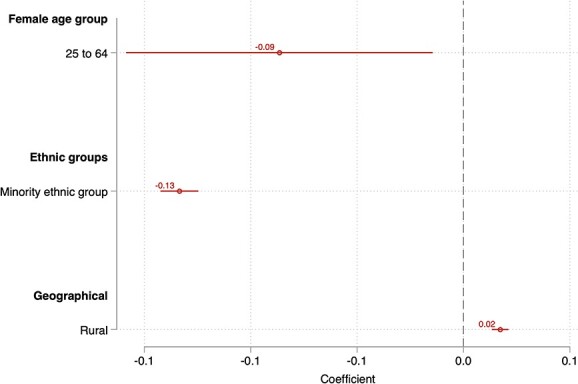
Association between patient characteristics and cervical screening rates. *Note*: 95% confidence intervals are presented.

Figure 3 presents results on patient characteristics from the correlated random effects regression. GP practices with higher proportions of women aged 25–64 years old and ethnicity minority patients have lower screening rates by 0.09 (95% CI: 0.16–0.01) and 0.13 (95% CI: 0.14–0.12), respectively. 

Our results are consistent with sensitivity checks (presented in Table A1 in the appendix) when we include smaller practices with ˂1000 patients, exclude the financial year 2020/21, and consider a balanced panel of GP practices.

## Discussion

### Main findings

Cervical screening rates have declined in recent years. It is therefore important to identify what characteristics are related to this decline with longitudinal data. We describe a 5-percentage point drop in screening coverage in England from 77% in 2013/14 to under 72% in 2021/22 which falls well below the NHS Cervical Screening program target of 80%.

Our study finds that GP workforce characteristics are important factors associated with screening coverage. Each additional FTE nurse (per 1000 patients) is associated with a nearly 2 percentage point increase in screening coverage. In the context of screening rates falling by an average of 5 percentage points across the study period, this is particularly striking. Patient list size is also a key factor in screening coverage, where an increase in patient list size (by 1000 patients) is associated with 0.11 percentage point decrease in screening coverage. Our results remain consistent when we exclude pandemic years 2020/21.

Measures of GP access and quality as assessed by patient satisfaction scores are weakly associated with screening coverage. This suggests that the ability of practices to meet the QOF clinical criteria (one of our practice quality measures) corresponds with higher screening rates.

Practices located in more income-deprived areas are associated with lower screening coverage. We find evidence for a monotonic reduction in screening rates across income deprivation quintiles when moving from the least to most deprived quintiles. Compared to the least deprived quintile, there is a 1.02 percentage point lower screening coverage associated with GP practices located in the most deprived quintile. We further found that practices with a higher proportion of women in the eligible age range and those with a greater proportion of ethnic minority patients have lower screening rates, on average.

### What is already known

Our findings support existing evidence from a cross-sectional study that finds certain GP practice characteristics are associated with cervical screening coverage.^23^ In particular, our study supports existing evidence^36^ that finds that the number of nurses in a GP practice is important for cervical screening coverage. It also supports the notion that cervical screening rates are associated with certain patient characteristics, including ethnicity.^16^

### What this study adds

Our study adds to existing evidence that describes falling screening rates in England prior to the pandemic, by being the first to provide a longitudinal assessment of screening rates between 2013/14 and 2021/22. We highlight that screening rates were falling even prior to the pandemic demonstrating that the COVID-19 pandemic is not the sole determining factor. An ongoing fall in screening coverage requires a contemporary interrogation of the drivers of low uptake, and interventions to improve it.

Utilizing panel data permitted the use of correlated random effects regression which had two advantages over the current evidence. First, we were able to assess within practice changes that represent year-to-year changes in practice characteristics on year-to-year changes in screening rates. Second, we were able to account for time invariant unobserved effects that may represent barriers to screening such as the accessibility or organizational structure of a general practice.

Our findings suggest that structural factors may play a more important role in screening coverage that previously acknowledged. Efforts to restore cervical screening rates need to consider the workforce context within GP practices as increases in both nurses and administrative staff are related to higher screening rates. These workforce issues are particularly relevant in primary care given the number of consultations in GP practices has been increasing in recent years but staff numbers have not risen in line with this.^37^ Our results emphasize that the type of workforce able to carry out the screening program is important, particularly the number of administrative staff that can assist with organizing screenings. This characteristic of a GP practice has not previously been considered in the cervical screening literature.

Further, the relation between deprivation and screening rates is important as more deprived areas typically have fewer GPs per 1000 patients than less deprived areas^38^ and face more acute workforce pressures.^39^^,^^40^

The advent of home-based self-sampling methods as an alternative to routine cervical screening^41^ may present opportunities to increase coverage by reducing the burden on primary care. Further research is needed to assess how self-sampling methods address barriers to cervical screening at the patient level and impact overall screening rates.

### Limitations of this study

We did not have access to individual level data on screening and socio-demographics, relying instead on practice level information. However, observing practices over time enabled us to use econometric panel data methods and therefore account for time in-variant unobserved factors that may be associated with screening.

Our variable capturing the ethnic composition of an LSOA was based on the 2011 census. If the ethnic composition of LSOA’s had substantially changed over our analyzed time period this may have impacted on our results.

Future work with access to nationally representative individual-level information would be able to understand how a variety of individual characteristics impact on cervical screening as well as the practice level factors we observed in the present study.

## Conclusion

Our study finds that cervical screening rates have been falling over time in English primary care. We identify GP practice workforce and patient characteristics that are related to lower screening coverage. Our findings demonstrate clear markers that can inform health decision-making priorities for cervical screening going forward. In particular, home-based self-sampling methods could provide an alternative route for screening, reducing the burden on primary care where staff shortages impair screening capacity. Furthermore, by improving access to cervical screening in socioeconomically deprived areas, self-sampling methods could help narrow the health inequity gap.

## Supplementary Material

Appendix_fdad275

## Data Availability

The data used in this study are publicly available online from the cited sources.

## Notes

**Sean Urwin,** Research Fellow

**Stephanie Gillibrand,** Research Fellow

**Jennifer C. Davies,** Clinical Research Training Fellow

**Emma J. Crosbie,** Professor
