# Supplementary material for: Factors associated with cervical screening coverage: a longitudinal analysis of English general practices from 2013 to 2022
Source: J Public Health (Oxf). 2023 Dec 26;46(1):e43–50. doi: 10.1093/pubmed/fdad275 (PMC10939411; doi:10.1093/pubmed/fdad275)
Supplement: Appendix_fdad275 [file appendix_fdad275.docx]

# Appendix

**Section A1.1:**

From 2013 to 2019 the cervical screening indicator applied to women between 25 and 65 years old. From 2019 this indicator was split into two with one indicator for the 25 to 49 years old age group and another for the 50 to 64 years old age group. This was to incorporate the different time intervals between screening invitations that varies by age group. We combined the post 2019 indicators to form one measure to ensure consistency across the period of analysis

The cervical screening indicator prior to 2018/19 covered women between 25 and 65 and required they had a screening every five years. This indicator, CS002, was worded as:

*“CS002: The percentage of women aged 25 or over and who have not attained the age of 65 whose notes record that a cervical screening test has been performed in the preceding 5 years”*

After 2018/19 the cervical screening indicator split into two separate indicators by age. The 25 to 49 years old required a screening every three and a half years whereas those 50 to 64 years old required a screening every five and a half year. The former indicator was worded as:

*“CS005 The percentage of women eligible for screening and aged 25-49 years at the end of the reporting period whose notes record that an adequate cervical screening test has been performed in the preceding 3 years and 6 months”*

The latter indicator was worded as:

*“CS006 The percentage of women eligible for screening and aged 50-64 years at the end of the reporting period whose notes record that an adequate cervical screening test has been performed in the preceding 5 years and 6 months”*

**Section A1.2:**

These eighteen ethnic groups are derived from five ethnicity metagroups: White (including White British, Irish, Gypsy or Irish traveller, White Other); Mixed (including White Black Caribbean, White Black African, White Asian, Other Mixed); Asian (including Indian, Pakistani, Bangladeshi, Chinese, Other Asian); Black (African, Caribbean, Other African) and Other (including Arab or any other ethnicity).

**Tables and Figures:**

Figure A1: Sample restrictions

Initial sample:

64353 GP practice-years

8020 GP practices

Practices with more than 1000 patients:

64009 GP practice-years

7953 GP practices

Practices with complete cases:

59218 GP practice-years

7881 GP practices

| Table A1: Sensitivity checks | | | | |
| --- | --- | --- | --- | --- |
|  | Main specification | Including small practices | Excluding financial year 2020/2021 | Balanced panel |
| **Practice characteristics:** | | | | |
| FTE Nurses per 1000 patients | 1.94*** | 2.15*** | 1.84*** | 2.52*** |
|  | (0.26) | (0.32) | (0.25) | (0.32) |
| FTE GPs per 1000 patients | 0.39** | 0.46** | 0.37** | 0.43** |
|  | (0.12) | (0.14) | (0.11) | (0.15) |
| FTE Admin per 1000 patients | 0.54*** | 0.43*** | 0.54*** | 0.61*** |
|  | (0.10) | (0.13) | (0.10) | (0.14) |
| List size (000’s) | -0.11*** | -0.10*** | -0.10*** | -0.12*** |
|  | (0.02) | (0.02) | (0.02) | (0.02) |
| Overall patient experience: very good | 0.02*** | 0.02*** | 0.02*** | 0.01*** |
|  | (0.00) | (0.00) | (0.00) | (0.00) |
| QOF clinical achievement | 0.10*** | 0.11*** | 0.10*** | 0.09*** |
|  | (0.01) | (0.01) | (0.01) | (0.01) |
| Patient satisfaction with opening hours: very good | 0.02*** | 0.02*** | 0.01*** | 0.02*** |
|  | (0.00) | (0.00) | (0.00) | (0.00) |
| 2^nd^ Income deprivation | 0.07 | 0.08 | 0.05 | 0.22 |
|  | (0.17) | (0.17) | (0.16) | (0.20) |
| 3^rd^ Income deprivation | -0.29 | -0.28 | -0.30 | -0.06 |
|  | (0.19) | (0.19) | (0.18) | (0.23) |
| 4^th^ Income deprivation | -0.79*** | -0.77*** | -0.79*** | -0.47 |
|  | (0.21) | (0.20) | (0.20) | (0.25) |
| 5^th^ Income deprivation (most deprived) | -1.02*** | -1.00*** | -0.98*** | -0.61* |
|  | (0.23) | (0.22) | (0.22) | (0.29) |
| **Patient characteristics:** | | | | |
| Proportion 25 to 64 years old | -0.09* | -0.07 | -0.02 | -0.11* |
|  | (0.04) | (0.04) | (0.03) | (0.05) |
| Minority ethnic group | -0.13*** | -0.14*** | -0.13*** | -0.12*** |
|  | (0.00) | (0.00) | (0.00) | (0.01) |
| Rurality | 0.02*** | 0.02*** | 0.02*** | 0.01*** |
|  | (0.00) | (0.00) | (0.00) | (0.00) |
| Constant | 53.92*** | 53.56*** | 52.82*** | 54.37*** |
|  | (2.17) | (2.11) | (2.14) | (3.83) |
| Practice-years | 59218 | 59346 | 52983 | 39420 |
| Practices | 7881 | 7917 | 7881 | 4380 |
| Note: All model estimated using correlated random effects regression. Standard errors in parenthesis clustered at the general practice level; *p<0.05; **p<0.01; ***p<0.001 | | | | |
